# Discovery of Hepatitis E and Its Impact on Global Health: A Journey of 44 Years about an Incredible Human-Interest Story

**DOI:** 10.3390/v15081745

**Published:** 2023-08-15

**Authors:** Mohammad Sultan Khuroo

**Affiliations:** Digestive Diseases Centre, Dr. Khuroo’s Medical Clinic, Srinagar, Jammu & Kashmir 190010, India; khuroo@yahoo.com; Tel.: +91-9906591044

**Keywords:** discovery, epidemic, sporadic, non-A, non-B hepatitis, hepatitis E virus, hepatitis E, pregnancy, transmission, acute liver failure, acute-on-chronic liver failure

## Abstract

The story of the discovery of hepatitis E originated in the late 1970s with my extreme belief that there was a hidden saga in the relationship between jaundice and pregnancy in developing countries and the opportunity for a massive epidemic of viral hepatitis, which hit the Gulmarg Kashmir region in November 1978. Based on data collected from a door-to-door survey, the existence of a new disease, epidemic non-A, non-B hepatitis, caused by a hitherto unknown hepatitis virus, was announced. This news was received by the world community with hype and skepticism. In the early 1980s, the world watched in awe as an extreme example of human self-experimentation led to the identification of VLP. In 1990, a cDNA clone from the virus responsible for epidemic non-A, non-B hepatitis was isolated. Over the years, we traversed three eras of ambiguity, hope, and hype of hepatitis E research and conducted several seminal studies to understand the biology of HEV and manifestations of hepatitis E. Many milestones have been reached on the long and winding road of hepatitis E research to understand the structure, biology, and diversity of the agent, changing the behavior of the pathogen in developed countries, and the discovery of a highly effective vaccine.

## 1. Introduction

“The path traversed in the discovery of hepatitis E has been a fabulous journey and I was blessed to be on board for a wonderful trip.”—Mohammad S. Khuroo [1].

Various discoveries in medicine have saved millions of lives and changed our view of the world. Each discovery has an underlying human-interest story related to complexities, missteps, near-misses, and ups and downs in their pursuit. The discovery of hepatitis E is one such fascinating story from one of the most remote regions of the world with extreme weather conditions and primitive healthcare and investigative facilities. The discovery of hepatitis E is a story of a strong will to grab a vital opportunity, which may come once in a lifetime for the lucky ones, and the application of a curious mind, creative thoughts, untiring efforts, and strong belief in oneself to find the answers. This story also highlights the important fact that discoveries do not necessarily require high-tech laboratories or institutions with cutting-edge research facilities but can be accomplished in very primitive situations, as described below [2].

## 2. Golden Age of Hepatitis Research

I grew up and was educated in an era that has been aptly called the “Golden Age of hepatitis research” [3]. I graduated from Govt. Medical College (GMC) Srinagar, Kashmir, India (MBBS: 1962–1968), and joined the Dept. of Medicine at GMC Srinagar for residency training. I completed my post-graduation in general medicine (MD General Medicine: 1968–1972) and started practicing general medicine at GMC Srinagar as a budding physician (Lecturer: 1972–1976). For further pursuits in knowledge and to enhance my clinical skills, I joined the Postgraduate Institute of Medical Education and Research (PGIMER), Chandigarh, for DM Gastroenterology (1976–1978). After completing my post-doctoral training, I returned to GMC Srinagar in June 1978 [4].

The knowledge on viral hepatitis during this period was rapidly evolving. From 1958 to 1964, Krugman et al. performed human transmission experiments at the Willowbrook State School in New York and proved that enterically transmitted infectious (type A) hepatitis and parentally transmitted serum (type B) hepatitis were caused by two immunologically distinct viruses [5]. The results of their study were conclusive but could not be repeated today [6]. Blumberg et al. (1965) made a breakthrough when they published a classic paper on the Australia antigen [7]. This subsequently led to the discovery of the hepatitis B virus (HBV) [8]. Feinstone et al. (1973) visualized hepatitis A virus (HAV) particles using immune electron microscopy (IEM) in stool extracts of patients with acute HAV infection [9]. Soon after, Alter et al. (1975) reported the occurrence of 8 out of the 12 cases of post-transfusion hepatitis in 108 multiply transfused open-heart surgery patients that were unrelated to HAV, HBV, CMV, and EBV. They termed it non-A, non-B hepatitis [10]. In 1978, two groups of investigators reported on a transmissible agent in post-transfusion non-A, non-B hepatitis [11,12]. It took several years before Choo et al. (1989) isolated cDNA from the post-transfusion non-A, non-B hepatitis genome and developed a serological test for its diagnosis [13]. Rizzetto et al. described the delta antigen–antibody system in carriers of the HBsAg in Torino, Italy, in the mid-1970s [14]. Based on transmission studies in chimpanzees at the United States National Institutes of Health (NIH), the delta antigen was recognized as a new and unique human RNA virus, the hepatitis D virus (HDV) [15]. As the letters A, B, C, and D were already assigned to various forms of viral hepatitis, it was the turn of the letter E to be assigned to the next form of viral hepatitis [16].

## 3. Enigma of Viral Hepatitis in India in the 1970s

In the late 1970s, during my fellowship, I observed that the emergency rooms and intensive care units of hospitals were always occupied to a considerable extent by pregnant women with jaundice and high mortality rates. Autopsies of the deceased patients uniformly revealed massive hepatic necrosis. I wondered and questioned myself as to why pregnant women in India should suffer from viral hepatitis, develop acute liver failure, and die. The late Prof. D. V. Datta at the PGIMER, Chandigarh, would often call me to the liver laboratory following the morning meeting and ask, “Sultan, why do pregnant women come with fulminant hepatitis and die?” He knew I did not know the answer and possibly wanted me to find out. Meanwhile, doing so, it became evident that the data published in various industrialized countries indicated that pregnancy does not increase the severity of disease and/or susceptibility to infection, and pregnant women appear to handle viral hepatitis without additional risk to themselves and the fetus [17]. It was concluded that the data on viral hepatitis and pregnancy from India and the Middle East were based on retrospective analyses of hospital admissions, relatively few cases, or patients in whom a definitive diagnosis was lacking because of inadequate documentation from laboratory tests and liver biopsies [18].

Additionally, there were several reports of large-scale waterborne epidemics of viral hepatitis from the Indian subcontinent, the Middle East, and Central Asia [19,20,21,22,23]. Among these, the Delhi epidemic of 1955–1956, which caused 29,300 cases and 266 deaths, was extensively studied by the Indian Council of Medical Research (ICMR), New Delhi, and the National Institute of Virology (NIV), Pune [24,25]. It was concluded that the epidemic was a classic example of hepatitis A, and the peak attack rate in young adults was due to waning immunity following previous exposure to HAV [3,26]. Unfortunately, the possibility of a human hepatitis virus other than HAV as the cause of this epidemic was not considered. The conclusions drawn from the Delhi epidemic led to a delay in the recognition of hepatitis E by over 25 years [27]. Sera from the Delhi epidemic of 1955–1956, stored at NIV Pune, were tested after the results of the Gulmarg Kashmir epidemic of 1978–1979 were known and were found to be related to non-A, non-B hepatitis [28].

## 4. Gulmarg Kashmir Epidemic of 1978–1979

The story of the discovery of hepatitis E originated in the late 1970s with my extreme belief that there was a hidden saga in the relationship between jaundice and pregnancy in developing countries; my passion for exploring this unholy alliance; and the occurrence of a massive epidemic of viral hepatitis that hit the Gulmarg Kashmir region in November 1978 [29]. A quick tour of the epidemic region revealed a major panic in the community and a tsunami-like situation, caused by the disease in young adults and deaths in pregnant women. The epidemic had affected around 200 villages with an estimated population of 600,000, situated on either side of a rivulet known as the Ningli Nallah. The Nallah originates from Alpather Lake, situated at the foot of the twin 4511 m tall Apharwat Peaks in the Pir Panjal mountainous range, 10 km from Gulmarg, Jammu, and Kashmir, India. After winding its way through the mountains and the valley for a distance of 31 km and covering an area of over 200 sq km, the stream flows into Wular Lake [30]. The stream serves multiple purposes for the population, including being a source of drinking water, ablution, public latrine sewage disposal, community garbage outlet, and site for utensil and linen washing, washing of animals, swimming for children, and fishing. Thus, the population drinks grossly polluted water from these contaminated streams.

To investigate the epidemic, several challenges included hard weather, difficult terrain, primitive healthcare, an unwilling team to join me, health risks to me and my family, and a lack of funding. I took it upon myself to live in the area, strengthen healthcare, support the community, and offered care at the doorstep. I build a large local survey team of 500 healthcare workers and spend my personal earnings on conducting the research without any external funding. Over six months, we conducted four innovative door-to-door surveys of affected villages with meticulous recording of the events using pen and paper and thorough analysis of the results. In each survey, every house was visited, and all household members filled out a questionnaire for clinical symptoms of hepatitis, a physical examination for icterus, and a urine dipstick for bilirubin. Blood and fecal samples were collected from patients with suspected hepatitis. Sera were tested for liver functions and serological markers and a diagnosis of viral hepatitis was made based on clinical and biochemical findings. Patients needing inpatient care were transported to a liver unit at the SMHS hospital affiliated with Govt. Medical College (GMC) Srinagar. A liver laboratory was established in GMC Srinagar to store and test all collected human samples.

The decision to visit, live, and work in the epidemic area was not without risks, as I had been warned by my colleagues. I and all my family members came down with the disease which amounted to icteric hepatitis with protracted transaminitis in one, anicteric disease in two, and seroconversion in two members. Today, I wish to say to my family members, “*Thank you for being there with me to support my belief and passion and suffer the pain and anguish in the path of human service and science*”. To my colleagues, the wise, who declined to join me, I say, “*Thank you for not joining me so that I could do it alone*”.

## 5. Epidemic Non-A, Non-B Hepatitis

**Figure 1:**
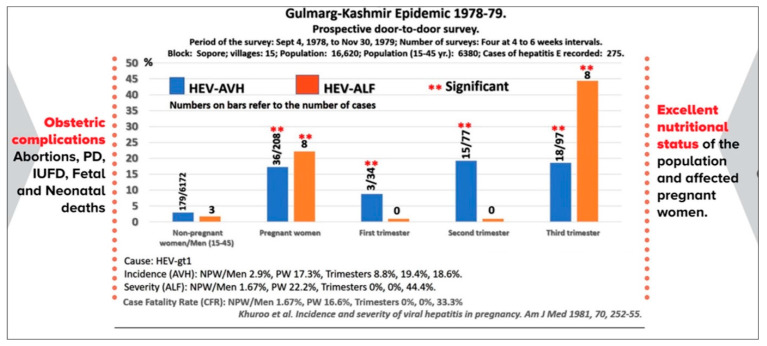
Incidence and severity of epidemic hepatitis E in pregnancy. Data were collected through a door-to-door survey of 15 villages during the Gulmarg Kashmir epidemic of 1978–1979. Pregnant women acquired HEV infections more often than men and non-pregnant women (15–45 years), in all three trimesters of pregnancy. Acute liver failure and consequent case fatality rates in HEV-infected pregnant women were higher than in HEV-infected men and non-pregnant women (15–45 years), selectively in the third trimester of pregnancy. HEV-infected pregnant women had high obstetric complications and had no evidence of malnutrition. The epidemic was caused by HEV-gt1.

The epidemiological data, the disease profile, the liver histology findings, and the serological results of the Gulmarg Kashmir epidemic of 1978–1979 were available by May 1979. A critical evaluation of the data was remarkable in its conclusions. The epidemic had unique features which led me, a young practicing physician at that time, to believe and announce the existence of a new disease, epidemic non-A, non-B hepatitis, caused by a hitherto unknown hepatitis virus [31]. The epidemic curve was unimodal, highly compressed, and lasted for 9 weeks (November–December 1978) with no secondary waves or cases. It was massive, involving 20,083 cases with 600 deaths. The disease pattern was unique as it affected young adults (15–45 years) with protracted cholestasis in a significant proportion (20%) of patients, acute liver failure in 4.36%, and a case fatality rate of 3.6%. Anicteric hepatitis occurred in 31% of the population. The mode of spread was fecally contaminated drinking water from Ningli Nallah. The liver histology was distinctively cholestatic with bile plugs and rosette formation of hepatocytes, minimal lobular inflammation, and disarray and expanded portal tract with features of acute cholangitis. Acute phase sera and fecal samples were non-reactive for acute markers of hepatitis A and B and all adults had antibodies and immunity to HAV from previous exposure. The epidemic adversely affected pregnant women causing a tale of death and devastation, a remarkable behavior of a unique pathogen, which has remained an enigma as of today [32] (Figure 1). Serial follow-up and 18-month assessment of patients with epidemic hepatitis revealed a self-limiting disease lasting for 4 to 6 weeks and no evidence of chronic liver disease, in contrast to post-transfusion non-A, non-B hepatitis [33,34]. Concurrently, a study of a large cohort of patients with sporadic acute non-A, non-B hepatitis revealed that the disease resembled the epidemic non-A, non-B hepatitis in the age of occurrence in adults, fecal–oral spread through person-to-person contact, increased prevalence and severity of disease in pregnant women, and no evidence of chronic hepatitis over a 6-month follow-up [35].

## 6. An Epoch-Making Event

The article in *The American Journal of Medicine* in 1980 [31] and the connoted term “*epidemic non-A, non-B hepatitis*” became an epoch-making event and was reproduced in the book “Classic Papers In Viral Hepatitis”, edited by Christine A. Lee and Howard C. Thomas with a forward from the late Dame Sheila Sherlock who called these papers a “Foundation laid by our predecessors” [36].

Stanley M. Lemon and Christopher M. Walker, while reviewing the article “Enterically Transmitted Non-A, Non-B Hepatitis and the Discovery of Hepatitis E Virus”, wrote “*Definitive evidence for an enterically transmitted non-A, non-B (ET-NANB) hepatitis agent followed a few years later. Mohammad S. Khuroo, a young gastroenterologist who had just completed his subspecialty training at the Institute of Medical Education & Research at Chandigarh, described a large, waterborne outbreak of hepatitis during the winter of 1978–1979 in the Baramulla district of the Kashmir valley, a remote, mountainous region in the north of India (Figure 1) (Khuroo 1980)*” [37].

Dr. Jay Hoofnagle while delivering a Great Teachers Lecture on “Hepatitis E: An emerging infectious disease” under the Clinical Centre Grand Rounds series at NIH, Bethesda, MD, remarked, “*Proof for a fifth type of viral hepatitis was first announced in 1980 when two groups of researchers (one from Kashmir and another from NAID’s Laboratory of infectious diseases) found that outbreaks of severe hepatitis in India were not due to hepatitis A or hepatitis B. These large epidemics had always been mentioned as “classical” examples of hepatitis A-infectious hepatitis caused by sewage contamination of the water supply, typically found in underdeveloped areas of the world (Asian subcontinent, sub-Saharan Africa, Central America). The fact that it wasn’t hepatitis A (or hepatitis B) was an eye-opener and led to its first, somewhat cumbersome name “epidemic non-A, non-B hepatitis*” [26].

Dr. Robert H. Purcell in his essay “*The discovery of hepatitis viruses” wrote, “Simultaneously with these studies, Mohammad Sultan Khuroo, another young gastroenterologist was studying a waterborne epidemic of hepatitis in the mountainous Kashmir region of North India. Hepatitis in Kashmir… Thus, the fifth recognized human hepatitis was discovered and initially called epidemic NANB hepatitis virus and subsequently designated as HEV*” [3].

Dr. Rakesh Aggarwal in his review “*Hepatitis E” wrote, “Hepatitis E was suspected for the first time in 1980 during a waterborne epidemic of acute hepatitis in Kashmir, India. In the 30 years since then, a small virus with a single-stranded RNA genome has been identified as the cause of the disease and named as hepatitis E virus (HEV)*” [38].

## 7. Breaking News and the Buzz

The breaking news of the discovery of a new disease, epidemic non-A, non-B hepatitis, and the possibility of the existence of a hitherto unknown hepatitis virus was accomplished in several ways. I presented the award-winning plenary session paper during the proceedings of the 20th Annual Conference of the Indian Society of Gastroenterology (ISG) held in Pune on 12 October 1979 [39]. The data were published in several journals of high impact which included two publications in *The American Journal of Medicine* “The Green Journal” [31,32], two letters in *The Lancet* [33,34], and a publication on sporadic non-A, non-B hepatitis in *The American Journal of Epidemiology* [35]. With the help of the late Nobel Laureate Baruch Blumberg, I was invited and presented a 3 min three-slide presentation on “Epidemic non-A, non-B hepatitis” during the plenary session of the First International Conference of Viral Hepatitis held in New York on 30 March 1981. From 23 March 1981 to 13 April 1981, I presented seminars and interacted with many legends at centers of excellence including NY Blood Center, NY Hospital-Cornell Medical Centre, Albert Einstein College of Medicine, and NIH, Bethesda, MD. This created a huge buzz and resulted in inquiries, invitations, support and collaboration from many national and international centers [40]. Several legends including Robert H. Purcell from NIH Bethesda, and Dr. Kunio Okuda and Dr. Masao Omata from Chiba Japan visited Kashmir and the epidemic area in 1980 and extended support and advice.

## 8. The Backlash

Along with the buzz, there was a strong backlash generated to question my claim of the existence of epidemic non-A, non-B hepatitis. Skeptics believed that this epidemic was caused by HAV and was not non-A, non-B hepatitis. They based their opinion on the results of the 1955–1956 Delhi epidemic which was considered a classic example of hepatitis A [3,26].

The backlash took several shapes. I faced watch-out alert calls and letters from friends and foes, one shall letter read as follows: “*Oct. 15, 1979. Dear Dr. Khuroo, Regarding the outbreak in your area, I discussed it with three legends in hepatology. They all suspect that this was a waterborne epidemic of hepatitis A. Dr. Kunio Okuda*”. There were several instances of an outpouring of extreme skepticism amounting to Pyrrhonism. A letter published in the *Lancet* on 12 July 1980, pages 365–66 [41], in response to one of our *Lancet* publications used derogatory words such as *“Appalled”, “Imagination rather than facts”, “Crude and pointless*”, etc. Unfortunately, the author of this letter formed his opinion and made statements without reading the two articles published earlier by our group in *The American Journal of Medicine* [31,32].

I fought skepticism with tolerance, courage, and support and collaborated with leading researchers, shipped thousands of sera and fecal samples to many international laboratories for testing, only to confirm that the sera lacked acute markers of HAV and HBV infections. So, by 1981, the whole world community was convinced that we were dealing with a new agent in the Gulmarg Kashmir epidemic of 1978–1979. On 28 April 1981, Dr. Robert Purcell wrote “*Dear Mohammad, … Your seminar was well received here at the National Institute of Health. I have heard many favorable comments about your presentation and the stimulating discussion that followed. I am more convinced now than ever that we are dealing with a new agent in these outbreaks of epidemic non-A, non-B hepatitis*”.

## 9. A Lost Opportunity

Based on the accumulated evidence that a hitherto unknown new agent existed in epidemic non-A, non-B hepatitis, I explored several possibilities for conducting animal or human transmission studies. A protocol for in-house transmission studies in Rhesus monkeys was drafted and submitted to the ethical committee for approval. Two life convicts had agreed for self-experimentation and legal and administrative permissions for this unusual endeavor were explored. I sought help from Dr. Alfred Prince at NY Blood Center; however, he declined and felt it carried a substantial risk to transport such highly infectious samples to the USA. Incidentally, he had reviewed my paper submitted to *The American Journal of Medicine* [31] for publication and had remarked that the paper was clear evidence for the existence of a fifth human hepatitis virus. Lastly, it was a matter of great satisfaction to collaborate with Robert H. Purcell at NIH to perform animal transmission studies on acute-phase human samples from the Gulmarg Kashmir epidemic of 1978–1979. We planned to inoculate primates with acute phase samples and serologically characterize any transmissible agent. Acute phase sera and fecal samples from the Gulmarg Kashmir epidemic of 1978–1979 reached Dr. Purcell’s lab in excellent condition on 11 August 1981. Unfortunately, he was not able to inoculate the specimen due to a shortage of chimpanzees in his laboratory at that time until Jan 1982. While all these activities were on track, what followed led me to believe that we were rather slow and had missed the bus.

## 10. Human Self-Experimentation: A Zest to Explore the Unknown

In the early 1980s, the world was in awe of an extreme example of self-experimentation by Dr. Mikhail Balayan, from the Institute of Poliomyelitis and Viral Encephalitides, Moscow, USSR [42,43]. In August 1981, Dr. Balayan along with Alexander Andjaparidze and Svetlana Savinskaya were investigating an outbreak of hepatitis among Soviet soldiers in the military camp in Afghanistan. Of the 620,000 Soviets who served in Afghanistan, 115,308 cases of hospitalization as a result of viral hepatitis were recorded [44]. Acute phase sera and stool were collected from 22 Soviet soldiers with non-A, non-B hepatitis similar to the Gulmarg Kashmir epidemic of 1978–1979. Of the 22 soldiers, 15 had a prior history of HAV infection in Feb 1981. The human samples were transported to a military base in Tashkent, Uzbekistan. A concentrated inoculum, prepared from feces of nine patients collected 1 to 4 days after the onset of jaundice and mixed with kefir (a fermented milk drink originating from the Caucasus], was ingested by Dr. Balayan. Dr. Balayan had IgG antibodies to HAV from a prior exposure. The news of this event was guarded and only a few persons knew about the remarkable human self-experimentation [37]. While he was involved in this experiment, he called me to enquire more about the Gulmarg Kashmir epidemic of 1978–1979 but did not divulge his intentions

Dr. Balayan returned to Moscow and on the 36th day of ingestion of the inoculum, developed a severe attack of hepatitic illness, which needed hospitalization for 4 days and supportive care.

His acute phase sera lacked markers of HAV and HBV infections. He started collecting and analyzing his own stool samples and on IEM, he identified novel 27–30 nm diameter virus-like particles (VLPs) in fecal samples collected between 28 and 45 days after his ingestion of the inoculum. The disease was transmitted to cynomolgus monkeys and resulted in elevated liver tests, liver injury on histology, excretion of VLP, and antibody responses in the animal model.

Balayan’s intentional self-experimentation engendered some controversy when details of the experiment were eventually reported. However, his rationale, when questioned subsequently, was that this was the most expeditious way to determine whether the outbreak was caused by an unknown virus that was antigenically distinct from HAV. I do agree with his interpretation and because of that, it defeated my multiple attempts to be the first to do transmission studies.

## 11. The Naming Ceremony of the Demon

Consequent to the identification of VLPs in epidemic non-A, non-B hepatitis, a time had come to name the demon and we faced a barrage of proposals and suggestions. However, the use of the alphabet “E” was the most appropriate for all reasons in the “*Namakarana*”. Hence, I proposed that the epidemic non-A, non-B hepatitis should be named “Hepatitis E” and the agent once characterized as “Hepatitis E virus (HEV)”, as the disease presents in *E*pidemics, it is *E*nterically transmitted, it is *E*ndemic in developing countries, and “*E”* virus should follow the known alphabet A, B, C, and D of viral hepatitis [16,45].

## 12. “Viruses-That-Were-Not-to-Be”

In the late 1980s, several spurious claims of isolating viruses “that-had-not-to-be” for epidemic non-A, non-B hepatitis were made. Investigators from Pasteur Institute, France, and the National Institute of Immunology, New Delhi, India, transmitted the disease to primates and isolated ‘Viral Hepatitis F’, a 27–34 nm double-stranded DNA virus as a causative agent of epidemic non-A, non-B hepatitis [46,47,48,49,50]. Uchida and colleagues [51] from Japan isolated an infective agent and found it to be a “silent” mutant of HBV. To counter these claims, thirty investigators met at NIH, Bethesda, MD, in April 1987 for a 2-day discourse on waterborne non-A, non-B hepatitis with advice from three legends, namely, the Nobel Laureate Dr. Albert Sabin (the inventor of the oral poliovirus vaccine (OPV)), the Nobel Laureate Dr. Baruch Blumberg (discoverer of the Australia antigen), and Dr. Stephen Feinstone (discoverer of HAV). The conference report heavily depended upon studies from Kashmir, Moscow, and CDC Atlanta, Georgia, USA, made several suggestions for further studies, and rejected all other claims about viruses causing non-A, non-B hepatitis [52].

## 13. Crossing the Hurdle

The years 1983 to 1990 were a period of setbacks in hepatitis E research as acute-phase human samples contained an extremely low amount of VLPs that were not sufficient for cloning and sequencing. Investigators had to examine more than 2000 stool samples to find one that contained a few VLPs. I believe this was the most frustrating 7-year halt in the story of hepatitis E. A surprise observation of finding large quantities of VLPs exceeding 1000/electron microscopic grid square from bile and not from stools of infected macaques by Daniel W. Bradley, CDC, ended the dilemma (unpublished observations). Virus-enriched gallbladder bile from cynomolgus monkeys infected with a third-passage Burmese isolate of HEV was used to construct recombinant complementary DNA (cDNA) libraries (in Lambda gt10). The libraries were screened through differential hybridization and one clone ET1.1 (approximately 1.3 kb) was identified that hybridized to an approximately 7.6-kilobase RNA species present only in infected cyno livers. This cDNA represented a portion of the genome of HEV [53]. Molecular cloning and sequencing of the entire 7.5 kb positive-strand RNA genome of the Burmese strain of HEV was completed shortly thereafter by the Genelabs group [54]. On this, Stanley M. Lemon and Christopher M. Walker while reviewing the discovery of hepatitis E wrote, “*Thus, by 1992, a decade after the discovery of the ET-NANB particle by Balayan* et al. *(1983) and a dozen years after Khuroo’s description of the epidemic in Kashmir (Khuroo 1980), HEV was firmly established within the panoply of human hepatitis viruses*” [37].

## 14. Seminal Studies from Kashmir

Over the years, our team at Kashmir traversed three eras of hepatitis E research namely the non-A, non-B era of ambiguity (1978–1983), the VLP era of hope (1983–1990), and the HEV era of hype (1990 onwards). Based on the activities of the field survey team, the liver unit, the liver lab, and the collaborations, we investigated 10 epidemics of hepatitis E from 1978 to 2013 involving around 55,563 icteric cases with 1772 deaths, studied thousands of cases of acute sporadic hepatitis and that of acute liver failure, and faced an ever-expanding pool of neonatal hepatitis E [55,56,57,58].

**Figure 2:**
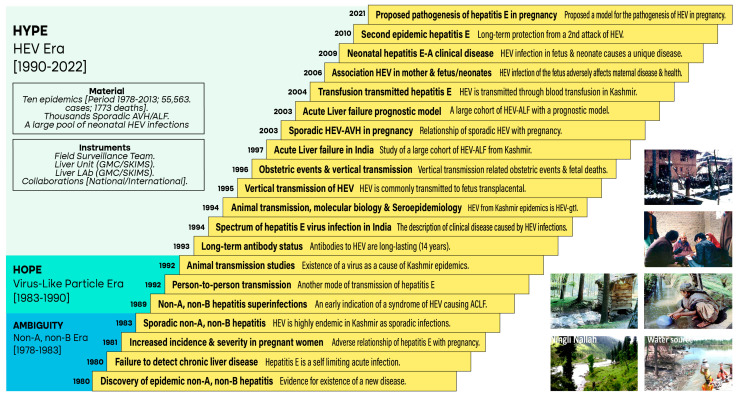
Seminal studies conducted in Kashmir on hepatitis E in three eras as defined. Each block identifies one published study protocol with the aim of the study (in bold) and consequent results. The year each study was published is shown. The right lower quadrant has 6 photographs taken of the region during the epidemic (1978–1979), showing the author standing in hard weather conditions (top picture), author along with the team conducting a door-to-door survey (second top), latrine sewage draining into waterways, (third left) and water collected from downstream (third right). The lower left image depicts Ningli Nallah flowing through the mountainous range and the lower right image depicts its multipurpose usage (sewage disposal, collection of drinking water, etc.). References include [31,32,33,34,35,59,60,61,62,63,64,65,66,67,68,69,70,71,72,73,74,75].

We conducted several seminal studies and published these in journals of repute to understand the biology of the pathogen, the hepatitis E virus, and manifestations of the human disease, hepatitis E (Figure 2) [31,32,33,34,35,59,60,61,62,63,64,65,66,67,68,69,70,71,72,73,74,75]. Apart from the discovery of hepatitis E as “epidemic non-A, non-B hepatitis” and its unique features [31,32,33,35,64], repeated epidemics allowed us to understand the dynamics of infection in the community and proposed a cohort phenomenon for the occurrence of the second and subsequent epidemics [27,45,74]. The disease was transmitted to rhesus monkeys and the Kashmir strain of HEV, characterized as HEV-gt1, was closely related to the Burmese stain of HEV [62,65]. The clinical course of HEV infection including serial liver test abnormalities, serologic responses, viremia, and virus shedding was studied [65]. Several clinical forms of hepatitis E disease were described, namely epidemic hepatitis E [31], sporadic hepatitis E [35], hepatitis E in pregnant women [69], hepatitis E acute liver failure [68,70], hepatitis E superinfections in chronic HBV carriers [60,64], and fetal and neonatal hepatitis E [73]. We established four routes of transmission of HEV infection in endemic areas [76] including water-borne spread due to fecally contaminated water supplies [31], person-to-person spread due to contact transmission [61], vertical transmission from infected pregnant women to the fetus and newborn [66], and transfusion-transmitted infection due to frequent short-lasting viremia in otherwise healthy donors [71]. With the continued support of field survey teams and repeat door-to-door surveys of the epidemic area in 1992, we established the persistence of antibodies after 14 years in the majority of the patients infected during the 1978 epidemic [63].

**Figure 3:**
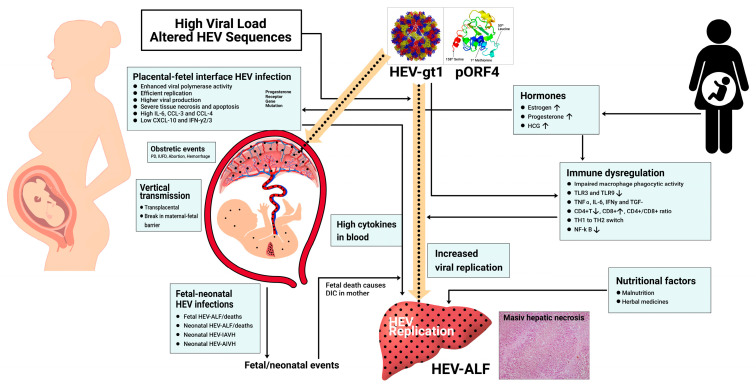
Proposed pathogenesis of adverse relationship between hepatitis E and pregnancy. See text for details. AIAVH = anicteric acute viral hepatitis, HEV-ALF = hepatitis E virus-related acute liver failure. IAVH=Icteric acute viral hepatitis.

Finally, we conducted extensive studies on the enigmatic relationship between hepatitis E and pregnancy (Table 1). We were the first to identify an increased incidence and severity of disease both in the epidemic [32] and sporadic disease [69] and the occurrence of explosive fulminant disease in the third trimester of pregnancy often with rapidly progressive cerebral edema, GI bleeding, DIC [70], and significant obstetric events, namely, abortions, intra-uterine fetal deaths, and premature deliveries. HEV infection in pregnancy is often transmitted to the fetus transplacentally and causes severe disease in the fetus and neonate which, in turn, adversely affects maternal disease [66,73,77]. On recognition of an additional ORF4 and the expression of a unique pORF4 in HEV-gt1 alone [78,79], I conducted an extensive study to show that the relationship between hepatitis E and pregnancy is HEV-gt1-specific [75]. Earlier, studies revolved around hormonal imbalances, immune dysregulation, and the poor nutritional status during pregnancy [80,81,82,83]. However, HEV-gt1, powered by pORF4, causes enhanced virus replication and consequent higher hepatic injury. In addition, HEV-gt1 infects the maternal–fetal interface and causes extensive necrosis and apoptosis, with the release of cytokines that flood the maternal blood with consequent higher liver injury [84,85,86]. The damage to the maternal–fetal interface leads to obstetric events and also breaks the barrier to cause fetal infections, severe fetal disease, and death. Severe fetal hepatitis and death often cause DIC in the mother, resulting in acute liver failure (Figure 3).

Recent studies from China have shown that HEV-gt4 causes uterine infections and injury and leads to obstetric events but does not increase the severity of the disease in the mother. Qian et al. [87] reported on the prevalence of HEV and its association with adverse pregnancy outcomes in 19,762 pregnant women from China. Adverse maternal outcomes included preterm birth, gestational diabetes, and pregnancy-induced hypertension syndrome. Vertical transmission resulted in low birth weight, macrosomia, fetal distress, spontaneous abortion, and fetal malformation. However, of the 111 pregnant women who were acutely infected by HEV, only 4 pregnant women (1 positive for HEV IgM antibody and 3 positive for HEV RNA) had elevated liver enzyme activities. None of the patients had clinical disease and no maternal deaths were reported. The authors believed that HEV-gt4 was responsible for mild liver injury in pregnant women [87,88,89].

Epidemics caused by HEV-gt2 in Mexico and Namibia did not report higher death rates in pregnant women [75,90,91]. However, a recent epidemic of viral hepatitis caused by HEV-gt2 in Burkina Faso reported more deaths in pregnant women [92]. In fact, 15 of 16 deaths occurred in pregnant or post-partum women. Few cases of HEV-gt3 infections reported in pregnant women have not been associated with severe disease or deaths [93,94,95]. As of today, there are no animal models that recapitulate all the clinical manifestations of vertical transmission of HEV as seen in humans [88,96,97,98,99,100].

## 15. Milestones in Hepatitis E Research

**Figure 4:**
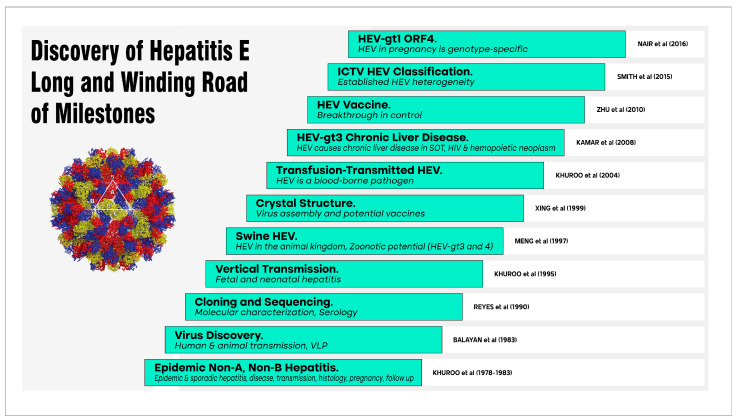
The long and winding road of milestones over the years in hepatitis E research. Each study was a turning point in understanding hepatitis E. Left lower quadrant depicts native HEV with T = 3 capsid morphology. References include [31,32,33,35,42,53,66,71,78,101,102,103,104,105]. Due to space constraints, only selected milestones are shown.

Over the ensuing years, many milestones have been achieved in the long, winding road of hepatitis E research in understanding the structure, biology, and diversity of the agent; the habitat and host of the agent in the animal kingdom; its evolution and population dynamics over time; the existing and changing behavior of the pathogen beyond our borders; and the discovery of a highly effective vaccine, HEV 239, which is a significant step to an ultimate cure and control of this pathogen (Figure 4) [31,32,33,35,42,53,66,71,78,101,102,103,104,105].

The hepatitis E story moved out of the developing countries with the isolation of a novel HEV isolate by Meng et al. [101] from domestic pigs which was closely related to, but distinct from, human HEV strains. It opened the Pandora’s box of the extreme heterogeneity of HEV [105] and its peculiar food-borne zoonotic potential to cause autochthonous infections in developed countries [106]. We were the first to report that hepatitis E virus infection may be transmitted through blood transfusions in an endemic area [71]. Soon, post-transfusion HEV infection was recognized as an important mode of transmission of HEV infection in industrialized countries [107,108]. Vertical transmission of HEV from infected mother to fetus [66,109] and the recent discovery of ORF4 in HEV-gt1 alone [78] set the path for understanding the pathogenesis of the relationship between hepatitis E and pregnancy [75]. Kamar et al. studied 14 cases of acute HEV-gt3 infection in patients receiving organ transplants. Eight developed chronic hepatitis E [103]. Chronic hepatitis caused by HEV-gt3 in the solid organ transplant, HIV, and hematopoietic neoplasm patients is a growing public health concern in the West [110]. The elucidation of the crystal structure of the capsid protein and the ability of selective amino acid residues to self-assemble into T = 1 (22 nm) empty virus-like particles set the path for vaccine development [102,104,111].

## 16. Hepatitis E Virus

**Figure 5:**
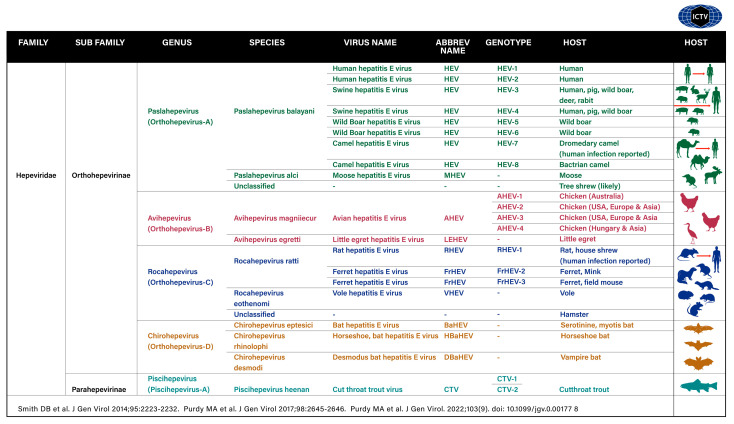
Hepatitis E virus: classification, nomenclature, and diversity. Members of the Hepeviridae family are assigned to two subfamilies, five genotypes, and ten species, and infect over a dozen species of hosts in the animal kingdom. Human infections occur in several ways and are depicted by red arrows.

Hepatitis E virus belongs to the family Hepeviridae, which has remarkable heterogeneity (Figure 5) [105,112]. Members of the Hepeviridae family are assigned to two subfamilies, five genotypes, and ten species, and they infect over a dozen species of hosts in the animal kingdom. Humans are infected by four genotypes in the genus *Paslahepevirus* (*Orthohepevirus A*). HEV-gt1and 2 are anthroponotic and only transmitted from humans to humans. HEV-gt3 and 4 are enzootic and infect pigs and a few other animals and infect humans through peculiar food-borne zoonotic transmission pathways [56,76]. A few human infections in SOT patients have been reported to occur from camels in Dubai by eating and drinking camel meat and milk [113] and from rats in Hong Kong through exposure to droppings in the environment [114,115].

**Figure 6:**
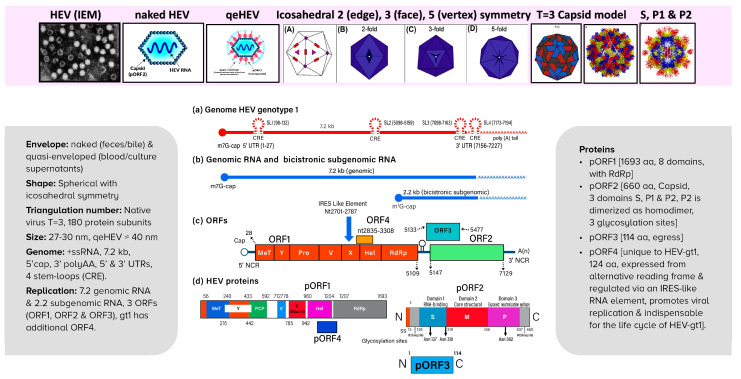
Structure of hepatitis E virus and genomic organization. For details, see the text in Hepatitis E Virus section.

Hepatitis E virus has a unique structure (Figure 6) [116,117,118]. HEV is a positive-sense, single-stranded RNA virus with a 7.2 kb genome; it is icosahedral in shape with a 2:3:5 symmetry, with a cap at the 5′ end, two UTRs, one at each 5′ and 3′ end. Four cis reactive elements form stem-loops across the genome, consisting of three partially overlapping open reading frames with defined functions. The genome replicates into a 7.2 genomic RNA and 2.2 subgenomic RNA. The native virus has a triangulation number of 3 with 180 protein subunits. Three unique features of the virus include a quasi-envelope HEV circulating in the blood [119,120,121], phenomenal three domains crystal structure of the capsid with P2 domain as a dimer [102,122], and an exceptionally important protein expressed only from ORF4 in genotype 1. pORF4 consists of 124 aa, promotes replication of the virus, is indispensable for the life cycle of genotype 1 and is possibly involved in the increased severity of infection in pregnancy [123,124].

**Figure 7:**
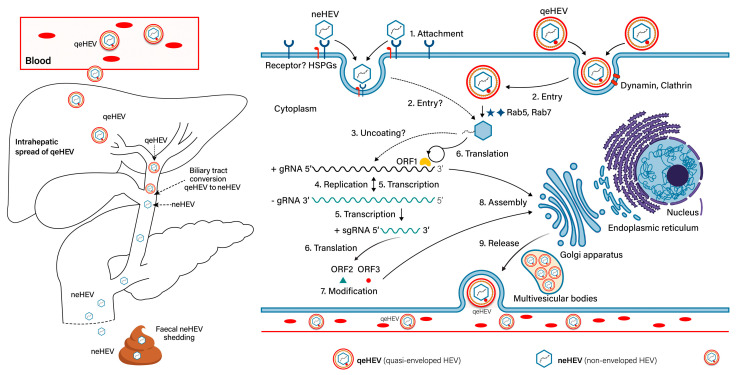
Proposed replication of hepatitis E virus. For details, see the text about hepatitis E replication.

The path followed by the virus from binding to egress from hepatocytes is depicted in Figure 7 [125,126]. Currently, the HEV life cycle remains poorly understood and there are large knowledge gaps in receptors and functional domains of ORF1. The distinct entry pathway of non-enveloped and quasi-enveloped virus particles are shown. While quasi-envelope particles circulate in the blood and are immune evasive, the particles lose their lipid coat in bile ducts and circulate as naked particles in the environment.

**Figure 8:**
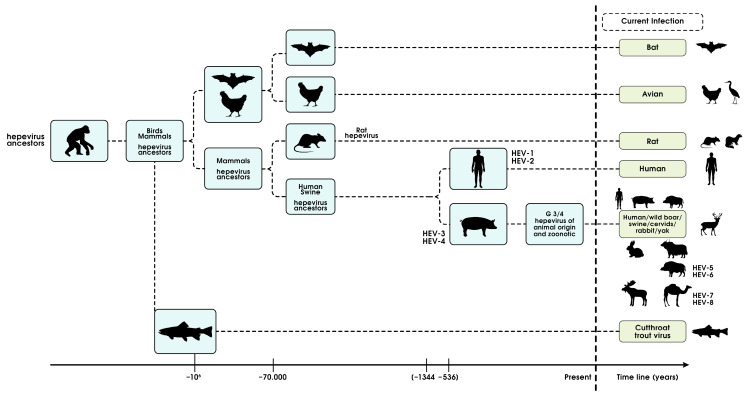
Evolutionary history of hepatitis E virus. The times to the most recent common ancestors (tMRCAs) were calculated using BEAST to conduct a Bayesian analysis of HEV. For details, see text on HEV evolution.

To commemorate the political and scientific controversy around the evolution of SARS-CoV-2, the evolution and population dynamics of human HEV are shown (Figure 8) [127,128,129,130]. Fortunately, humans seem to be farther from bats [131] and chickens and nearer to rodents according to cross-species transmission evaluations. Around 5 to 13 centuries “Before-Present (BP)”, human HEV evolved through natural selection into anthroponotic HEV-gt1 and 2, affecting only humans, and enzootic HEV-gt3 and 4, affecting pigs and related animals and transmitted to humans through a peculiar food-borne pathway. The spread of HEV-gt4 to Japan coincided with the export of pigs from India. HEV-gt4 underwent a recent expansion to China to nearly replace the long-existing HEV-gt1 strain [132,133,134,135].

## 17. Hepatitis E

**Figure 9:**
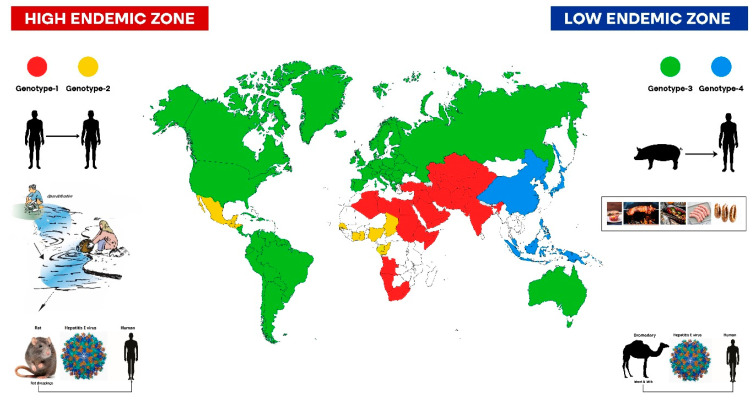
Global distribution of hepatitis E virus.

Hepatitis E has a global distribution (Figure 9) [55,56,136]. HEV-gt1 and 2 only infect humans, with contaminated water as the transport vehicle, and cause hyperendemic and endemic disease in resource-poor countries [55,76,137]. There are unique ways in which public water supplies are contaminated by sewage; however, open defecation continues to be a major public health issue in the Indian sub-continent. In contrast, HEV-gt3 and 4 infect and exist heavily in the swine population, and cross-transmission of the virus across deer, boar, and pig occurs and exposure to humans follows several pathways [138]. However, most autochthonous human infections occur by the peculiar habit of eating raw or undercooked pig meat, liver, and sausages [139]. Thus, the availability of clean water and proper cooking of pig meat are major weapons to fight against and eliminate hepatitis E.

HEV is an emerging global infection of major public health importance (Table 2) [140,141]. According to the latest WHO report, every year, there are an estimated 20 million HEV infections worldwide, leading to an estimated 3.3 million symptomatic cases of hepatitis E. Hepatitis E caused approximately 44, 000 deaths in 2015 [137,142]. HEV-gt1 and, to a lesser extent, HEV-gt2 are major public health problems in endemic areas of the world through repeated massive waterborne epidemics, each involving hundreds and thousands of cases [143,144,145]. HEV in these regions is the most common cause of acute sporadic hepatitis, acute liver failure, and acute-on-chronic liver failure. There is an enormous load of hepatitis E in pregnancy and fetal/neonatal HEV infections [146,147]. Of late, there are declining trends in these figures due to improvements in sanitation in India. Hepatitis E is no longer a disease of developing countries! HEV-gt3 and 4 infections are the most successful zoonotic viral diseases in human history and cause zoonotic food-borne autochthonous infections in industrialized countries. With the availability and application of HEV diagnostics, the existence of hepatitis E in several other forms has been established including transfusion-transmitted infections, inducing screening programs by several countries, a large pool of HEV-gt3 chronic liver disease in solid organ transplant, HIV, and hematopoietic neoplasm patients, masquerading as drug-induced liver injury and a series of syndromes caused by extrahepatic manifestations of HEV [138,148,149].

The development of accurate diagnostic assays for the detection of HEV infection remains challenging and many outstanding issues remain (Table 3) [150]. The serological tests have issues of sensitivity and specificity. NAAT testing, essential in several scenarios, is only available in specialized laboratories and these tests need to be developed, standardized, and made widely available for day-to-day practice.

Apart from the modulating immunosuppressive ribavirin, monotherapy has been a cornerstone for managing chronic hepatitis E in SOT patients (Table 4) [110,151]. Pegylated interferon alfa and Sofosbuvir are alternative options in the case of ribavirin treatment failure. The use of ribavirin is contraindicated in pregnancy; however, its use in HEV-ACLF is worth testing in selected cases. There have been attempts to evaluate alternative drugs in treating difficult patients with HEV-gt3 chronic hepatitis [152].

**Figure 10:**
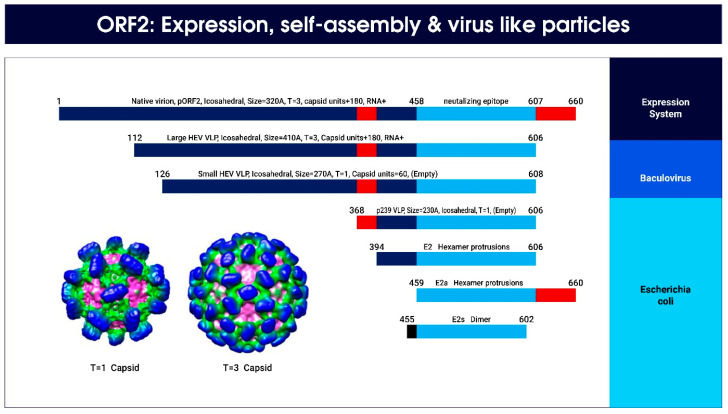
Hepatitis E ORF2 expression, self-assembly, and virus-like particles with native HEV (T = 3) and assembled VLP (T = 1). HEV ORF2 proteins with different lengths have been expressed and purified in several systems in order to determine their particle-forming properties [154]. The native HEV particles have 660 aa with T = 3 icosahedral symmetry and 180 units of the ORF2 protein, encircling HEV RNA [102]. In the baculovirus insect cell system, the HEV capsid protein can self-assemble into either a large or a small VLP [156]. In the E. coli system, the shortest proteins, termed E2 and E2a, form hexamer protrusions and E2s form dimers [157,158]. Adding 26 amino acids toward the N-terminus of the pE2 peptide results in higher-order assembly structure p239 (368–606 aa), which is highly immunogenic [159]. Due to space constraints only two expression systems are depicted and selective assembled VLP are shown.

The discovery of the hepatitis E vaccine, HEV 239 Hecolin, has been a breakthrough in the history of hepatitis E and has been accomplished through expression, virus assembly, and formation of highly immunogenic VLPs from 239 aa of ORF2 protein expressed in an E. coli system (Figure 10) [102,104,111,154,155]. The vaccine has successfully completed phase II and III trials and is highly efficacious with long-lasting protective effects. Its availability and application in other regions of the world, particularly the Indian subcontinent, is being actively pursued.

## 18. Challenges

What are our challenges in the control and cure of this global human pathogen? These include further development, standardization, and availability of diagnostic tests; a better understanding of the perplexing biological and epidemiological behavior of this pathogen; the development of treatment strategies and management policies for those with severe infections and acute liver failure; and the development and implementation of effective preventive strategies, especially HEV vaccines.

## 19. Conclusions

To conclude, my journey of 44 years presents hard work, persistence, belief in oneself, and honesty of purpose. It meant spending days on the snowbound, dilapidated roads of those villages and feeling the pain and anguish of the sufferers. It needed courage to stand on one’s feet and face the inherent bias and skepticism, and listening to the wise. I have spent nights awake with pen and paper to write what I believed was true and what to uncover. The journey was treacherous, hard, and full of failures and some successes. However, the end was pleasant and rewarding, for discoveries do not come without a price to be paid [56].

## Figures and Tables

**Table 1:** Relationship of hepatitis E with pregnancy in endemic areas (data collected from studies conducted in Kashmir [32,35,66,68,69,70,75,77]).

Epidemic HEV-AVH and pregnancy [Khuroo et al. 1981]
During epidemics, pregnant women acquire HEV infection 8 times more than non-pregnant women and men (15–45 years), being 8.8%, 19.4%, and 18.6% in the three trimesters. Around one-fourth (22.2%) of HEV-infected pregnant women develop ALF as against 1.6% in HEV-Infected non-pregnant women and men (15–45 years), being 0%, 0%, and 44.4% in the three trimesters respectively. CFR of pregnant women is 16.6% as against 1.67% in non-pregnant women and men (15–45 years), being 0%, 0%, and 33.3% in the three trimesters respectively.
Sporadic HEV-AVH and pregnancy [khuroo et al. 1983 and 2003]
HEV infection is the cause of sporadic AVH in around 85.5% of pregnant women as against 41.5% in non-pregnant women and men (15–45 years). ALF develops in around 69.2% of pregnant women with HEV infection as against 10.0% in non-pregnant women and men (15–45 years).
HEV-ALF and pregnancy [khuroo et al. 1997 and 2003]
Around 95.8% of ALF in pregnant women are caused by HEV as against 41.1% in non-pregnant women (15–45 years). HEV-ALF in pregnant women is an explosive disease with short PEP, high occurrence of cerebral edema, and DIC. CFR in HEV-ALF is 51.9% and was significantly less than CFR in non-HEV-ALF (84.2%). Pregnancy per se or duration of pregnancy did not adversely affect prognosis
Obstetric complications in pregnancy with HEV infection [khuroo et al. 1981, 2003 and 2006]
Pregnant women with HEV infection had a higher occurrence of obstetric complications than those with non-HEV infection. Obstetric complications in HEV-infected pregnant women include preterm labor, antepartum hemorrhage, intrauterine fetal deaths, abortions, and neonatal deaths.
Vertical transmission of HEV and its implications [khuroo et al. 1995, 2006, 2009, and 2021]
Vertical (transplacental) fetal/neonatal HEV infection is reported to occur in 33–100% of pregnant women with HEV infection. About 15% of HEV-infected fetuses either die in utero or abort. Liver histology shows massive hepatic necrosis. Around half of the HEV-infected neonates develop ALF and present with hypoglycemia, hypothermia, and death. Of the remaining around one-fourth develop self-limiting acute hepatitis (30.4%) or anicteric hepatitis (22%). No chronic viremia, hepatitis, or chronic liver disease. Mothers who deliver HEV-infected babies early (within 4 days of disease) survive more than those who deliver late. DIC in mothers with HEV-ALF occurred exclusively when the fetus/neonate had an HEV infection with massive hepatic necrosis.

**Table 2:** Hepatitis E: global scenario.

	High-Incidence Endemic Zone [Low Resource Countries]	Low-Incidence Endemic Zone [Industrialized Countries]
Virus	HEV-gt1 and 2	HEV-gt3 and 4
Regions affected	HEV-gt1: Central and Southeast Asia and the Middle East HEV-gt2: northern and sub-Saharan Africa and Mexico	HEV-gt3: Europe, USA, South America, Australia, and Russia HEV-gt4: China, Japan, and many countries in SE Asia
Host/ Reservoir	Only humans; no animal reservoir	HEV-gt3: pig, boar, deer, and rabbit HEV-gt4: pig and boar
Mode of transmission	Polluted drinking water * Person-to-person contact transmission Vertical transmission € Transfusion-transmitted ¶ No transmission from pigs (HEV-gt4) to humans	Zoonotic food-borne transmission ** Slurry *** Transfusion-transmitted ¶ Ingestion of camel milk/meat (camel HEV-gt7) Exposure to rat droppings (rat HEV)
Clinical disease	Epidemic AVH (nearly all cases) Sporadic AVH (30–50% sporadic infections) ALF (One-third of cases of ALF) ACLF (One-third of cases of ACLF) Neonatal HEV-AVH and ALF € Extra-hepatic disease £	Icteric AVH (elderly people and alcoholics) Masquerades as DILI ¥ Chronic hepatitis and cirrhosis Ω Extra-hepatic diseases £
High-risk groups	Pregnancy Cirrhosis	Elderly people and alcoholics SOT, HIV, and hemopoietic NP patients (HEV-gt3)
Estimated load	Latest WHO report: 20 million infections Estimated 3.3 million symptomatic cases and 44,000 deaths (in 2015)	The estimated load is not known; occurs as: Autochthonous infections µ Transfusion-transmitted HEV ¶ Chronic hepatitis and cirrhosis Ω DILI ¥ Extra-hepatic diseases £
Trends	Years 1990–2013: Improved sanitation caused a declining trend in mortality	China: Recent expansion of HEV-gt4 to China, replacing HEV-gt1

* Pollution occurs under diverse settings—open defecation; open multipurpose water supplies; following Monsson/floods; leaking city water pipes; faulty sewage disposal. ** Infections occur by consuming raw undercooked game meat, raw pig liver, and Figatelli sausage. *** Slurry causes contamination of water and subsoil and infection through strawberries and mollusks. € Vertical (transplacental) transmission of HEV from infected mother to fetus is known to occur in 33–100% of pregnant women. Vertically transmitted fetal HEV infections and HEV infections in newborns have a wide spectrum of manifestations including severe fetal disease and death; syndrome of ALF in newborns presenting as hypothermia, hypoxia, hypoglycemia, and death within 24 to 48 h of birth; neonatal acute icteric and anicteric hepatitis. ¶ Transfusion-transmitted HEV infections occur due to donor blood HEV RNA viremia. The HEV viremia in donor blood is estimated at 1 in 745 donor samples (Japan), 1 in 671 donor samples (Germany), and 1 in 2848 donor samples (England). Estimated transfusion-transmitted HEV infections in the year 2013: 80,000 to 100,000 in England, 1600 to 5900 in Germany; genotype: HEV-gt3. £ Extra-hepatic diseases reported in developing countries include pancreatitis, Guillain–Barre syndrome, cholecystitis, etc. HEV-gt3-specific neurological syndromes pose a problem in clinical practice in England and Europe. Other extrahepatic syndromes reported from such countries include pancreatitis, hemolysis, cryoglobulinemia, myocarditis, and reports of male infertility in China (gt4). ¥ A substantial proportion of drug-induced liver disease in the West is due to HEV-gt3. Ω Large series of HEV-gt3-related chronic hepatitis and cirrhosis in SOT, HIV, and hemopoietic NP patients reported from Europe and England. µ Reports of autochthonous infections include ECDC surveillance report from 2005 to 2015: 21,018 cases, 37 small outbreaks, 80% in Germany, France, and England, 98.5% autochthonous infections, 28 deaths; genotype: HEV-gt3. IASR surveillance report 2005 to 2013: 562 cases, 86.3% autochthonous infections, source of infection was raw pig and game meat; genotype: HEV-gt4. *References have been cited in text under hepatitis E.*

**Table 3:** HEV infection: diagnosis.

Test (Method)	Uses (Comments)
IgM anti-HEV (ELISA and ICT-POCT)	Acute infection (assay varies in performance; poor performance in immune disorders; negative for re-infections).
IgG anti-HEV (ELISA and ICT-POCT)	Seroprevalence; natural protection; vaccine efficacy (assay varies in performance).
HEV RNA (NAT)	Acute infection in immunosuppressed patients; confirm chronicity; check anti-viral response, and donor blood screening (viremia is short lasting; in-house assays vary in performance).
HEV antigen (ELISA)	Diagnose acute infection (81% concordance with HEV RNA, cost-effective).
HEV genotyping (sequencing)	Study virus strain (important in understanding the pathogenesis of HEV infection).
ICT-POCT = immunochromatographic point-of-care technique; ELISA = enzyme linked immunoassay; NAT = nucleic acid testing.	

**Table 4:** Drug therapy for hepatitis E virus infection and algorithm for treatment of chronic HCV.

Class (Drug)	Effect on Viral Replication	Clinical Use
Calcineurin inhibitors (Cyclosporine, Tacrolimus)	Stimulates replication Increases viral load Promotes viral persistence	Reduce dose
mTOR inhibitors (Rapamycin, Everolimus)	Stimulates replication Increases viral load	Reduce dose
Antimetabolite immunosuppressants (Mycophenolate mofetil)	Inhibits replication helps viral clearance	Continue drug
Guanosine analog (ribavirin)	Inhibits replication Helps viral clearance	Primary drug for therapy
Cytokines (PEGylated interferon α)	Inhibits replication Helps viral clearance	Indicated in ribavirin failure
Nucleoside analog (Sofosbuvir)	Inhibits replication in vitro A clinical trial demonstrated that sofosbuvir was not effective on HEV [153]	The role is unclear. May need further trials
Definition of chronic HCV: HEV RNA-reactive for more than 3 months.		
Treatment algorithm of chronic HCV: Reduction of immunosuppression (if possible), effective in around 30% of cases. Ribavirin as a primary drug (600 mg per day); therapy for 3 months, effective in >90% of cases. Non-responders: Use PEGylated IFN α in liver transplant patients. May add Sofosbuvir (results inconclusive).		

## Data Availability

Not applicable.

## Abbreviations

HEV = hepatitis E virus; HAV = hepatitis A virus; HBV = hepatitis B virus; HCV = hepatitis C virus; HDV = hepatitis D virus; AVH = acute viral hepatitis; gt = genotype; ALF = acute liver failure; ACLF = acute-on-chronic liver failure; IEM = immune electron microscopy; VLP = virus-like particle; cDNA = complementary DNA; ORF = open reading frame; pORF = protein of ORF; ISG = Indian Society of Gastroenterology; GMC = Government Medical College, Srinagar, Kashmir, India; PGIMER = Post-graduate Institute of Medical Education & Research, Chandigarh, Punjab, India; ICMR = Indian Council of Medical Research, New Delhi, India; NIV = National Institute of Virology, Pune, Maharashtra, India.

## Declaration

I submit that any reference to an individual or institution in this article is circumstantial and I hold no malice against anybody.
